# Thrombosis-related characteristics of pregnant women with antithrombin deficiency, protein C deficiency and protein S deficiency in Japan

**DOI:** 10.1186/s12959-024-00581-z

**Published:** 2024-02-08

**Authors:** Takao Kobayashi, Kazuko Sugiura, Toshiyuki Ojima, Mariko Serizawa, Kyuya Hirai, Eriko Morishita

**Affiliations:** 1 Department of Obstetrics and Gynecology, Hamamatsu Medical Center, 328 Tomitsuka-Cho, Chuo-Ku, Hamamatsu, Shizuoka, 432-8580 Japan; 2 Faculty of Nursing, Department of Women’s Health, Nursing and Midwifery, Fukuoka Prefectural University, 4395 Ita, Tagawa, Fukuoka, 825-8585 Japan; 3 Department of Community Health and Preventive Medicine, Hamamatsu University School of Medicine, 1-20-1 Handayama, Chuo-Ku, Hamamatsu, Shizuoka, 431-3192 Japan; 4 Department of Obstetrics, Gynecology and Family Medicine, Hamamatsu University School of Medicine, 1-20-1 Handayama, Chuo-Ku, Hamamatsu, Shizuoka, 431-3192 Japan; 5 Department of Laboratory Sciences, College of Medical, Pharmaceutical and Health Sciences, Kanazawa University, 5-11-80 Kodatsuno, Kanazawa, Ishikawa, 920-0942 Japan

**Keywords:** Antithrombin deficiency, Pregnant women, Protein C deficiency, Protein S deficiency, Thrombophilia

## Abstract

**Background:**

We previously conducted a primary survey of pregnant women with hereditary thrombophilia based on national surveillance in Japan, but did not examine their thrombosis-related characteristics. Antithrombin (AT) deficiency, protein C (PC) deficiency and protein S (PS) deficiency are the major types of hereditary thrombophilia in Japan.

**Methods:**

We examined their detailed information related to thrombosis, and evaluated peripartum outcomes in comparison with control data obtained from the Japan Society of Obstetrics and Gynecology.

**Results:**

Definite or probable AT deficiency, PC deficiency and PS deficiency were observed in 80, 50, and 317 pregnancies, respectively, from 2014 to 2018 in Japan, with prevalence rates among total deliveries of 0.011%, 0.007%, 0.044%. The number of pregnancies with AT, PC and PS deficiency might have been as many as 27, 17 and 108 every year if complete answers had been provided. In the peripartum period of current pregnancies, 27.5% of women with AT deficiency, 28.0% with PC deficiency and 13.2% with PS deficiency developed thrombosis (*p* < 0.001 vs. control). Pregnant women with AT and PC deficiency were more susceptible to thrombosis than those with PS deficiency (*P* < 0.01). Of the thromboses, 92.3% occurred during pregnancy, 62.8% at less than 15 gestational weeks. The earliest onset of thrombosis was 5 gestational weeks. Prophylactic anticoagulation significantly prevented the onset of both antepartum and postpartum thrombosis (*p* < 0.0001). The rate of recurrent pregnancy loss in women with low PC or PS activities was significantly higher than in controls (*p* < 0.0001); however, it is unknown whether recurrent pregnancy loss is related to hereditary PS deficiency. There seem to have been few serious maternal or fetal/neonatal complications due to placental insufficiency related to a hypercoagulable state other than growth restriction.

**Conclusions:**

This survey revealed the thrombosis-related characteristics of pregnant women with hereditary thrombophilia in Japan. We suggest prophylactic anticoagulation to prevent maternal or fetal/neonatal complications.

## Background

Antithrombin (AT) deficiency, protein C (PC) deficiency and protein S (PS) deficiency are the major types of hereditary thrombophilia in Japan. Factor V (FV) Leiden mutation and prothrombin 20210G > A, which are major pathological variants of thrombophilia in Western countries, have still not been reported in Japanese people [1–4]. Hereditary thrombophilia is a syndrome in which severe thrombosis may develop in people under 40 years old [1, 5–9]. It often causes early onset of venous thromboembolism (VTE) or repeated recurrences of VTE, which can be fatal [10, 11]. VTE is usually either pulmonary embolism (PE) or deep vein thrombosis (DVT), but there are also cases of thrombosis in unusual sites. When a woman with thrombophilia becomes pregnant, thrombosis may develop during pregnancy or after delivery [1, 12–14]. It is also hypothesized that thrombophilia may cause placental insufficiency as a result of placental vascular thrombosis. The resulting abnormalities in the uteroplacental circulation may cause recurrent pregnancy loss (RPL), intrauterine growth restriction, placental abruption, preeclampsia (hypertensive disorder of pregnancy: HDP), fetal demise, preterm birth, and other problems [15–17].

Recently, we investigated the number of patients with hereditary thrombophilia and their peripartum management in Japan between 2014 and 2018, and estimated that there could have been up to 29 pregnant women with AT deficiency, 23 with PC deficiency and 151 with PS deficiency in each of those years [18]. Since that investigation was a primary survey, however, detailed information such as the time of thrombophilia diagnosis, family history or past history of thrombophilia, onset of thrombosis during pregnancy or after delivery, thromboprophylaxis, pregnancy complications or bleeding risk from anticoagulation, and fetal/neonatal complications was unknown. In this study, we examined the abovementioned detailed information related to thrombosis in pregnant women with AT deficiency, PC deficiency and PS deficiency, and evaluated their peripartum outcomes compared to control data on pregnant women in Japan.

## Methods

### National surveillance questionnaire

A national surveillance of pregnant women with hereditary thrombophilia between January 1, 2014 and December 31, 2018 was conducted in 2019. The design of the study was previously reported in detail [18]. Among the 415 institutions to which we sent a paper surveillance questionnaire, 243 responded to the questionnaires. Among the 243 responding institutions, 102 institutions had 599 cases of thrombophilia in the primary survey. In this study, we obtained the following detailed information in each case of thrombophilia; (1) maternal background data, such as family or past history of thrombosis, time of diagnosis, activity and/or antigen level at diagnosis, outcomes of past pregnancies including recurrent pregnancy loss (RPL), preterm delivery, hypertensive disorder of pregnancy (HDP), placental abruption, and other conditions, (2) maternal data for the current pregnancy, such as age, height, body weight, mode of delivery, spontaneous abortion or preterm delivery, (3) outcomes of current pregnancy, such as onset of thrombosis, HDP, placental abruption, major bleeding, and other conditions, (4) prophylactic anticoagulation in current pregnancy, (5) antithrombin supplementation in current pregnancy, and (6) neonatal data such as body weight, small for gestational age (SGA), sex, Apgar score, asphyxia, deformity and other outcomes. All personal information in this study was anonymized.

### Inclusion and exclusion criteria

In the primary survey, hereditary thrombophilia was diagnosed according to the criteria of each institution, based especially on low AT, PC and PS activities. The lower limits of the adult reference values are generally about 70% for AT, 55–70% for PC, and 60–70% for PS without the use of warfarin [1]. In this study, however, we established the strict inclusion and exclusion criteria for pregnant women with hereditary thrombophilia shown in Table 1, to distinguish patients with definite or probable thrombophilia. Thus, pregnant women who did not satisfy the inclusion criteria, or who met the exclusion criteria, were excluded from this study. In particular, women with low PS activity measured only during pregnancy had to be excluded from the PS deficiency group unless they satisfied inclusion criteria other than low PS activity.

**Table 1:** Inclusion and exclusion criteria for pregnant women with hereditary thrombophilia

**Inclusion criteria**
1. Plasma activity levels of AT, PC, or PS when not pregnant are below the lower limits of the adult reference values (generally about 70% for AT, 55–70% for PC, and 60–70% for PS) without the use of warfarin. Individuals with low PS activity measured only during pregnancy must satisfy at least one of inclusion criteria 3–8. In these cases, PS deficiency can be diagnosed if free protein S antigen levels in the second and third trimesters are less than 30% and less than 24%, respectively 2. Japanese pregnant women who delivered from Jan 1, 2014 to Dec 31, 2018 3. One or more family members exhibiting the same symptoms as the patient 4. Past history of early onset thrombosis (age 40 or below) 5. Repeated recurrence of thrombosis 6. Thromboses in unusual sites 7. New onset of thrombosis during current pregnancy or after delivery 8. Patients with diagnosis confirmed by gene analysis Inclusion criteria 1 and 2 must always be met regardless of items of 3–8
**Exclusion criteria**
1. Thrombophilia other than AT, PC, and PS deficiency 2. Antiphospholipid syndrome, systemic lupus erythematosus, platelet abnormalities, vascular disorders, blood flow obstruction, paroxysmal nocturnal hemoglobinuria, malignant tumor and other conditions that tend to cause thrombosis 3. Pregnant women who delivered in 2019 (in cases of pregnant women who were managed in 2018)

### Comparison observational study

The control data on pregnant women in Japan used in this study were obtained from the perinatal database of the Perinatology Committee, Japan Society of Obstetrics and Gynecology (JSOG) for the period between January 1, 2014 and December 31, 2018. These data were collected from general hospitals and perinatal medical centers that tended to manage high-risk deliveries throughout Japan. All obtained control data were anonymized. The abovementioned detailed information from each case of thrombophilia in the primary survey was compared to the control data, and statistical differences were calculated.

### Statistical analysis

The differences in maternal or neonatal background data and outcomes between definite or probable patients with thrombophilia and the control data on pregnant women in Japan were assessed using Pearson’s chi-squared test. The outcomes of current pregnancy and the effect of peripartum prophylactic anticoagulation were also analyzed using Pearson’s chi-squared test. Only the effect of prophylactic anticoagulation among women with a previous history and/or family history of thrombosis was analyzed using the Mantel–Haenszel method. Statistical analysis was done using SPSS version 25. In these analyses, a *p*-value of less than 0.05 indicated statistical significance.

### Details of ethics approval

The national surveillance of pregnant women with hereditary thrombophilia was approved by the Ethics Committee of Kanazawa University (approval number 890–1) and Hamamatsu Medical Center (rapid approval number 78, 2018). The comparison observational study was approved by the Ethics Committee of Kanazawa University (approval number 1210–1) and Hamamatsu Medical Center (approval number 2021–3-095) and the Ethics Committee of the JSOG (approval number 131). This study was performed in compliance with the Declaration of Helsinki. Consent was not obtained, but all presented data were anonymized and there is no risk of individual identification. Although the ethics committees decided that informed consent was not required due to the retrospective nature of the study, we adopted the opt-out method.

## Results

### Number of pregnant women with definite or probable thrombophilia and past maternal background data

In counting the number of cases of thrombophilia, each time a woman delivered during the study period was counted as one case. It was also counted as one case when one woman had combined thrombophilia deficiencies. However, the number of cases of thrombophilia was counted as one case in instances of multiple pregnancy. As a result, the numbers of cases of AT deficiency, PC deficiency and PS deficiency that met the inclusion criteria but not the exclusion criteria from 2014 to 2018 were 80, 50, 317, respectively (Table 2). Since 725,405 women delivered after 22 gestational weeks (GW) at the responding institutions [18], the prevalence of AT deficiency, PC deficiency and PS deficiency was 0.011%, 0.007%, 0.044%, respectively. Among them, 49 cases of AT deficiency, 12 cases of PC deficiency and 42 cases of PS deficiency were in women with a family history of thrombosis. Twenty-seven cases of AT deficiency (all VTE), 17 cases of PC deficiency (14 cases of VTE) and 48 cases of PS deficiency (37 cases of VTE) were in women with a past history of thrombosis before the current pregnancy.

**Table 2:** Maternal background data

		AT deficiency	PC deficiency	PS deficiency	Total			AT deficiency	PC deficiency	PS deficiency	Total
Number of cases		80	50	317	447	Number of pregnancies	0	14^‡^	9*	40^§^	63^§^
Combined deficiency (yes)		4	11	13	28		1	37*	20	76^#^	133
Family history of thrombosis only (yes)		29	7	31	67		2	21	8	77^‡^	116^‡^
Past history of thrombosis only (yes)		7	12	37	56		≥ 3	8	13*	122^§^	143^§^
Both family and past history of thrombosis (yes)		20	5	11	36	Number of deliveries	0	36	27	146	209*
Gene analysis (yes)		24	10	11	45		1	34	19	128*	181^‡^
Time of diagnosis	Before current pregnancy	51	38	257	346		2	9	4	37	50
	During current pregnancy	29	12	60	101		≥ 3	1	0	4*	5*
Mean activity at diagnosis (mean ± SD, %)		47.6 ± 11.4	48.4 ± 10.5	40.3 ± 13.7	42.8 ± 13.4	Number of spontaneous abortions	0	37	24	108^§^	169^§^
Mean antigen level at diagnosis (mean ± SD, %)		64.4 ± 30.2	44.8 ± 9.9	55.4 ± 20.0	55.0 ± 20.9		1	7	1^‡^	9^§^	17^§^
Type I deficiency		26	26	36	88		2	3	9^§^	38^§^	50^§^
Type II deficiency		8	0	71	79		≥ 3	2	4^#^	64^§^	70^§^
Type unknown		46	24	210	280	Complications of past pregnancy		27^nd^	22^nd^	164^nd^	213^nd^

*AT* Antithrombin, *PC* Protein C, *PS* Protein S, Type I deficiency is classified as a quantitative abnormality in which both the activity and antigen levels are decreased. Type II deficiency is classified as a qualitative abnormality in which the activity level decreases even though the antigen level is normal. The obtained effective number of pregnancies and deliveries among controls were 1,181,079 and 1,179,592, respectively. No mark means not significant vs. control, *: *P* < 0.05 vs. control, ‡: *P* < 0.01 vs. control, #: *P* < 0.001 vs. control, §: *P* < 0.0001 vs. control, nd: not determined

As shown in Table 2, there was one case of AT and PC deficiency, 3 cases of AT and PS deficiency and 10 cases of PC and PS deficiency. Mean enzyme activity at diagnosis was 47.6 ± 11.4% in AT deficiency, 48.4 ± 10.5% in PC deficiency, and 40.3 ± 13.7% in PS deficiency. The percentage of women with more than 2 and more than 3 spontaneous abortions (RPL) were 6.3% and 2.5% in those with AT deficiency (not significant [ns] vs. control), 26.0% and 8.0% in those with PC deficiency (*p* < 0.0001 and *p* < 0.001, respectively), and 32.2% and 20.2% in those with PS deficiency (both *p* < 0.0001), respectively. Among them, only 9 women (one with AT deficiency, 2 with PC deficiency and 6 with PS deficiency) had a family and/or past history of thrombosis. Furthermore, 2 women with AT deficiency, one with PC deficiency and 52 with PS deficiency were diagnosed before their current pregnancy through screening of activities in women with RPL.

### Maternal background data of current pregnancy

The maternal data for the current pregnancy are shown in Table 3. Age at delivery was 30.5 ± 4.68 years old in those with AT deficiency, 34.8 ± 4.81 in those with PC deficiency and 34.5 ± 4.96 in those with PS deficiency. The rates of vaginal delivery, scheduled cesarean section (C-section) and emergency C-section excluding abortions were 76.6%, 16.9% and 6.5% in those with AT deficiency, 50.0%, 25.0% and 25.0% in those with PC deficiency and 63.3%, 20.8% and 16.0% in those with PS deficiency, respectively. The rate of C-section in PC deficiency was significantly higher (*p* = 0.018) and that in AT deficiency was marginally significantly lower (*p* = 0.051) compared to the controls. The rate of emergent C-section in those with PC deficiency was significantly higher (*p* < 0.05) and that in those with AT deficiency was significantly lower (*p* < 0.05) compared to the controls. The rates of spontaneous abortion and preterm delivery were 2.5% and 13.0% (ns) in those with AT deficiency, 2.0% and 10.4% (ns) in those with PC deficiency and 0.3% and 12.5% (ns) in those with PS deficiency, respectively.

**Table 3:** Maternal background data of current pregnancy

			AT deficiency *n* = 80	PC deficiency *n* = 50	PS deficiency *n* = 317	Total *n* = 447	Control *n* = 1,171,651
Age at delivery (mean ± SD, years old)			30.5 ± 4.68	34.8 ± 4.81	34.5 ± 4.96	33.7 ± 4.92	32.4 ± 5.40
Height at delivery (mean ± SD, cm)			158.4 ± 5.59	158.1 ± 15.7	158.0 ± 7.93	158.6 ± 5.53	157.8 ± 5.75
Body weight in early pregnancy (mean ± SD, kg)			51.2 ± 7.96	56.0 ± 8.80	52.3 ± 7.55	52.5 ± 7.85	53.7 ± 9.70
Body weight at delivery (mean ± SD, kg)			58.3 ± 8.01	63.2 ± 9.05	60.2 ± 8.47	60.2 ± 2.43	63.3 ± 9.81
GW at delivery (mean ± SD, weeks)			37.6 ± 2.43	38.4 ± 7.05	37.9 ± 3.63	37.8 ± 3.27	38.1 ± 2.50
Body mass index at delivery	< 18.5		14	2*	27^§^	43^§^ (10.5%)	188,452 (18.2%)
	18.5 ≤ < 25.0		50	36	243^§^	329^§^ (80.2%)	717,337 (69.2%)
	25.0 ≤		10	6	22^‡^	38* (9.3%)	131,204 (12.7%)
Mode of delivery	Vaginal	Single	59	24*	198	281 (62.9%)	765,266 (65.3%)
		Multiple	0	0	0	0 (0.0%)	9348 (0.8%)
	Cesarean section (scheduled)	Single	11	12	60	83 (18.6%)	178,654 (15.2%)
		Multiple	2	0	5	7 (1.6%)	35,956 (3.1%)
	Cesarean section (emergency)	Single	5*	12*	46	63 (14.1%)	153,850 (13.1%)
		Multiple	0	0	4	4 (0.9%)	28,577 (2.4%)
Preterm delivery (22 ≤ < 37 GW)			10	5	39	54 (12.1%)	163,576 (14.0%)
Spontaneous abortion (< 22 GW)			2 (13, 19 GW)	1 (19 GW)	1 (20 GW)	4 (0.9%)	no data
Artificial abortion (< 22 GW)			1	1	3	5 (1.1%)	no data

*AT* Antithrombin, *GW* Gestational weeks, *PC* Protein C, *PS* Protein S. The obtained effective number of deliveries among controls was 1,171,651. No mark means not significant vs. control, *: *P* < 0.05 vs. control, ‡: *P* < 0.01 vs. control, §: *P* < 0.0001 vs. control, Totals do not add up due to missing values

### Outcomes of current pregnancy

**Fig. 1:**
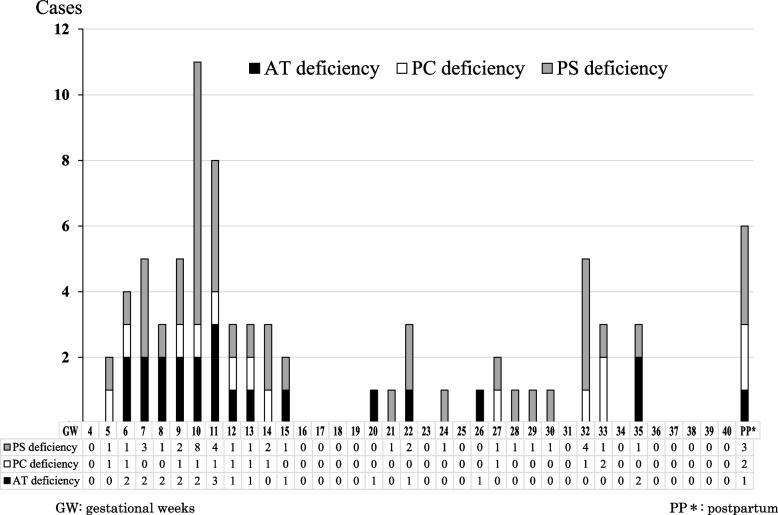
Number of thrombosis cases by gestational weeks

As shown in Table 4, thrombosis developed in 22 cases (27.5%) of AT deficiency, 14 cases (28.0%) of PC deficiency and 42 cases (13.2%) of PS deficiency. The incidence of thrombosis in women with all these thrombophilia was significantly higher than in the controls (*p* < 0.0001); however, the incidence in cases of PS deficiency was significantly lower than that in cases of AT and PC deficiency (*p* < 0.01). DVT (59 cases), PE (5 cases) and DVT with PE (10 cases) were the most common (*p* < 0.0001 vs. control). Two cases of cerebral vein thrombosis and 2 cases of arterial thrombosis occurred. The number of thrombosis cases by GW is shown in Fig. 1. The earliest onset of DVT was 5 GW in cases of PC deficiency and PS deficiency, and that of PE was 8 GW in a case of AT deficiency. Of the thromboses that occurred, 92.3% (72/78) occurred during pregnancy, and 62.8% (49/78) occurred at less than 15 GW. Only 6 cases of thrombosis (7.7%) occurred after delivery. Among pregnancy complications related to a hypercoagulable state, there were no significant differences in placental abruption, HDP and HELLP syndrome compared to the control cases (Table 4).

**Table 4:** Outcomes of current pregnancy

		AT deficiency *n* = 80	PC deficiency *n* = 50	PS deficiency *n* = 317	Total *n* = 447	Control *n* = 1,171,651
Thrombosis	Deep vein thrombosis (DVT)	15	12	32	59 (13.2%)	889 (0.08%)
	Pulmonary embolism (PE)	1	0	4	5 (1.1%)	218 (0.02%)
	DVT + PE	5	1	4	10 (2.2%)	no data
	Cerebral vein thrombosis	1	0	1	2 (0.4%)	no data
	Arterial thrombosis	0	1	1	2 (0.4%)	no data
	Total (%)	22^§^ (27.5%)	14^§^ (28.0%)	42^§^ (13.2%)	78^§^ (17.4%)	1107 (0.09%)
Placental abruption		0	0	2	2 (0.4%)	10,036 (0.86%)
Hypertensive disorder of pregnancy (HDP)		1	3	15	19 (4.3%)	68,342 (5.83%)
HELLP (homolysis, elevated liver enzymes, and low platelet count) syndrome		0	0	2	2 (0.4%)	3447 (0.29%)
Atonic bleeding		3	0	3^§^	6^§^ (1.3%)	71,603 (6.11%)
Gestational diabetes mellitus		2	2	13	17* (3.8%)	71,542 (6.11%)
Premature rupture of the membranes		0	0	10^§^	10^§^ (2.2%)	5251 (0.45%)
Breech presentation		1	1	2^§^	4^§^ (0.9%)	74,781 (6.38%)
Placenta previa/accreta		1	0	5^‡^	6^‡^ (1.3%)	5251 (0.45%)

*AT* Antithrombin, *PC* Protein C, *PS* Protein S. In thrombosis among women with thrombophilia, there were significant differences between AT deficiency and PS deficiency, and between PC deficiency and PS deficiency (both *p* < 0.01). However, there was no significant difference between AT deficiency and PC deficiency. The obtained effective number of deliveries among controls was 1,171,651. No mark means not significant vs. control, *: *P* < 0.05 vs. control, ‡: *P* < 0.01 vs. control, §: *P* < 0.0001 vs. control

### Prophylactic anticoagulation in current pregnancy

Antepartum, intrapartum and postpartum prophylactic anticoagulation in the current pregnancies are shown in Table 5. In antepartum prophylactic anticoagulant management, unfractionated heparin (UFH) combined with low dose aspirin (LDA) was given in 59.4% of cases of PS deficiency (*p* < 0.0001) and in 40.0% of cases of PC deficiency (*p* < 0.01), which was significantly higher than in cases of AT deficiency (7.1%).

**Table 5:** Prophylactic anticoagulation in current pregnancy

		AT deficiency *n* = 80	PC deficiency *n* = 50	PS deficiency *n* = 317	Total *n* = 447
Antepartum	Prophylactic anticoagulant therapy	42	30	175	247
	Unfractionated heparin (UFH) only	37	18	70	125
	UFH combined with LDA^※^	3	12	104	119
	Others^#^	2	0	1	3
	No occurrence of thrombosis	38	28	174	240
	Onset of thrombosis	4	2	1	7*
	No prophylactic anticoagulant therapy	38	20	142	200
	No occurrence of thrombosis	21	10	104	135
	Occurrence of thrombosis	17	10	38	65**
Intrapartum	UFH only	19	11	43	73
Postpartum	Prophylactic anticoagulant therapy	72	35	166	273
	UFH/low moleculer weight heparin (LMWH) only	17	12	93	122
	UFH/LMWH combined with warfarin	46	18	62	126
	Others^§^	9	5	11	25
	No occurrence of thrombosis	71	34	166	271
	Occurrence of thrombosis	1	1	0	2*
	No prophylactic anticoagulant therapy	8	15	151	174
	No occurrence of thrombosis	8	14	148	170
	Occurrence of thrombosis	0	1	3^$^	4**

*AT* Antithrombin, *PC* Protein C, *PS* Protein S. ※: In the antepartum period, there was significantly more frequent use of UFH combined with LDA both in women with PS deficiency (*p* < 0.0001) and PC deficiency (*p* < 0.01) than in women with AT deficiency. However, there was no significant difference in women with PS deficiency and PC deficiency. *: All of these 9 cases had a previous history and/or family history of thrombosis. **: Twenty-five of 69 cases had a previous history and/or family history of thrombosis. $: All of these 3 cases developed thrombosis after stopping postpartum prophylactic anticoagulant therapy. #: Others includes one case of warfarin and 2 cases of LMWH. §: Others includes 19 cases of warfarin only, 5 cases of DOAC and one case of UFH combined with LDA. The prophylactic anticoagulation used the most was 10,000 units/day of UFH. The dose of LDA was 80–100 mg/day and that of LMWH was 2000–4000 units/day. Although it is not an anticoagulant therapy, LDA alone without UFH was used in 90 cases (3 cases of AT deficiency, 6 cases of PC deficiency and 81 cases of PS deficiency) in the antepartum period

In a group of women receiving antepartum prophylactic anticoagulation (247 cases), thrombosis occurred in 7 cases (4 cases of AT deficiency, 2 cases of PC deficiency and one case of PS deficiency). In contrast, in a group of women receiving no prophylactic anticoagulation (200 cases), thrombosis occurred in 65 cases (17 cases of AT deficiency, 10 cases of PC deficiency and 38 cases of PS deficiency). In a group of women receiving postpartum prophylactic anticoagulation (273 cases), thrombosis occurred in 2 cases (one case of AT deficiency and one case of PC deficiency). On the other hand, in a group of women receiving no prophylactic anticoagulation (174 cases), thrombosis occurred in 4 cases (one case of PC deficiency and 3 cases of PS deficiency). Prophylactic anticoagulation significantly prevented the onset of both antepartum and postpartum thrombosis (*p* < 0.0001). Among women with a previous history and/or family history of thrombosis (159 cases; 56 cases of AT deficiency, 24 cases of PC deficiency and 79 cases of PS deficiency) (Table 2), thrombosis occurred in 9 cases in a group receiving peripartum prophylactic anticoagulation, and thrombosis occurred in 25 cases in a group receiving no peripartum prophylactic anticoagulation. Again, prophylactic anticoagulation significantly prevented the onset of peripartum thrombosis (*p* < 0.0001).

### Antithrombin supplementation in current pregnancy

Peripartum prophylactic AT supplementation in current pregnancies in patients with AT deficiency is shown in Table 6. The most common amount of AT supplementation was 1500 ≤  < 3000 units. In antepartum management, supplementation was performed in 67.5% of cases, in intrapartum management supplementation was performed in 60.0% of cases, and in postpartum management, supplementation was performed in 36.3% of cases.

**Table 6:** Antithrombin supplementation in current pregnancy

Dose of antithrombin supplementation (Units/week)	1500	1500 < < 3000	3000 ≤	Depended on activity	Total (%)
Antepartum	14	17	19	4	54 (67.5)
Intrapartum	21	25	2	0	48 (60.0)
Postpartum	12	9	7	1	29 (36.3)

### Neonatal background data

Neonatal background data are shown in Table 7. Body weights of term newborn babies were 2784 ± 413.0 (mean ± SD) g in women with AT deficiency, 2827 ± 546.1g in women with PC deficiency and 2852 ± 499.0g in women with PS deficiency (ns vs. control). The incidences of SGA infants among full-term deliveries were 17.4% in women with AT deficiency (*p* < 0.05 vs. control), 14.0% in women with PC deficiency (ns) and 11.5% in women with PS deficiency (ns). As for deformity or neonatal complications other than SGA, outcomes were better than in the controls.

**Table 7:** Neonatal background data

		AT deficiency *n* = 77	PC deficiency *n* = 48	PS deficiency *n* = 313	Total *n* = 438	Control *n* = 1,178,337
Body weight of term newborn babies (mean ± SD, g)		2784 ± 413.0	2827 ± 546.1	2852 ± 499.0	2837 ± 290.1	2861 ± 566.3
Sex	Male	38	25	168	231 (52.5%)	606,354 (51.3%)
	Female	37	23	149	209 (47.5%)	574,518 (48.7%)
Apgar score	1 min (mean ± SD)	8.1 ± 1.2	7.9 ± 1.3	8.0 ± 1.3	8.0 ± 1.3	8.0 ± 1.4
	5 min (mean ± SD)	9.1 ± 0.6	9.1 ± 0.7	9.0 ± 0.9	9.0 ± 0.8	8.9 ± 1.1
Full term delivery (37 ≤ < 42 GW)	Large for gestational age (LGA)	0	1	2	3 (0.8%)	8818 (0.9%)
	Appropriate for gestational age (AGA)	57	36	245	338 (86.4%)	909,762 (89.7%)
	Small for gestational age (SGA)	12*	6	32	50* (12.8%)	95,434 (9.4%)
Preterm delivery (22 ≤ < 37 GW)		10	5	43	58 (13.2%)	162,229 (13.8%)
Multiple pregnancy		2	0	9^‡^	11^#^ (2.5%)	74,310 (6.3%)
Non reassuring fetal status		0^‡^	4	8^#^	12^§^ (2.7%)	90,742 (7.7%)
Neonatal asphyxia (5 min Apgar score < 7)		2*	1*	6^§^	9^§^ (2.1%)	125,653 (10.7%)
Deformity		2	1	7	10 (2.3%)	15,990 (1.4%)
Neonatal death		0	0	2	2 (0.5%)	7115 (0.6%)
Other complications		3	5	12	20 (4.6%)	nd
Mean enzyme activity of newborn (mean ± SD, %)		51.5 ± 43.1	13.0 ± 0.00	58.6 ± 32.1	36.2 ± 23.9	nd

*AT* Antithrombin, *GW* Gestational weeks, *PC* Protein C, *PS* Protein S. The obtained effective number of newborn babies (≥ 22 GW) among controls was 1,178,337. No mark means not significant vs. control, *: *P* < 0.05 vs. control, ‡: *P* < 0.01 vs. control, #: *P* < 0.001 vs. control, §: *P* < 0.0001 vs. control, nd: not determined. The number of preterm deliveries of women with PS deficiency included 4 cases of twins. The percentages of LGA, AGA and SGA are the respective percentages among the number of full term deliveries. As there were no cases of over term delivery (≥ 42 GW) in thrombophiliac patients, control data are not shown. Abortion cases were excluded from these data

## Discussion

Definite or probable AT deficiency, PC deficiency and PS deficiency were observed in 80, 50, and 317 pregnancies, respectively, from 2014 to 2018 in Japan, with prevalence rates among total deliveries of 0.011%, 0.007%, 0.044%. In the peripartum period of current pregnancies, 27.5% of women with AT deficiency, 28.0% with PC deficiency and 13.2% with PS deficiency developed thrombosis. Of the thromboses that occurred, 92.3% occurred during pregnancy, and 62.8% occurred at less than 15 GW. Prophylactic anticoagulation significantly prevented the onset of peripartum thrombosis (*p* < 0.0001). There seem to have been few serious complications due to placental insufficiency other than growth restriction (SGA infants).

AT, PC and PS deficiency are the major types of thrombophilia in Japanese people [1, 5–9]. In this study, 4 cases of AT deficiency, 17 cases of PC deficiency and 126 cases of PS deficiency in the results of primary study [18] were excluded. Most of the cases that were excluded were those with low PS activity measured only during pregnancy, and those with plasma activity levels that did not meet the inclusion criteria (Table 1). If 100% of institutions had responded in this surveillance, it is estimated that there may have been up to 137 cases of AT deficiency, 85 of PC deficiency and 542 of PS deficiency during these 5 years. In other words, there could have been up to 27 cases of AT deficiency, 17 of PC deficiency and 108 of PS deficiency every year. National surveys by Ministry of Health, Labour and Welfare research groups and academic societies have indicated that there are about 2000 individuals with AT, PC, and PS deficiencies throughout Japan. The number of patients in whom these deficiencies newly develop annually is estimated to be less than 100 among neonates and infants, and about 500 among adults [19–25]. Thus, the estimated annual cases of pregnant women with hereditary thrombophilia in this study would seem to be reasonable. The prevalence of combined deficiencies in this survey was 3.2% (14/433). A nationwide survey in Japan conducted in 2009 revealed 12 patients with combined deficiencies among 183 patients with thrombophilia (combined AT and PC deficiencies; 4 cases, AT and PS deficiencies; 3 cases, PC and PS deficiencies; 5 cases), for a prevalence of 6.6% [26]. Furthermore, 5 cases of double mutations in PC and PS genes were reported among 55 patients with genetic mutations (9.1%) out of 173 Japanese patients with DVT [27]. Accordingly, the prevalence of combined deficiencies in cases of thrombophilia seems vary from 3 to 9% in Japan.

Three hundred and forty-six cases (77.4%) of thrombophilia (63.8% of AT deficiency, 76.0% of PC deficiency and 81.1% of PS deficiency) were diagnosed before the current pregnancies; however, the rates of gene analysis were low (30.0% of AT deficiency, 20.0% of PC deficiency and 3.5% of PS deficiency). Accordingly, the prevalence of type I or type II deficiency in each thrombophilia is not clear. Judging from activities and antigen levels, there seems to be more type I than type II in AT and PC deficiency, and more type II than type I in PS deficiency (Table 2). These tendencies would seem to be compatible with the fact that the PS gene polymorphism (PS p.K196E; type II deficiency) is seen in about 2% of the Japanese population [28–31].

Hereditary thrombophilia is a syndrome in which severe thrombosis may develop before the age of 40 [1, 5–9]. Therefore, when a woman with thrombophilia becomes pregnant, thrombosis may develop during pregnancy or after delivery. Accordingly, prophylactic anticoagulation is recommended as long as thrombotic risks continue [1, 12–14]. In this survey, 27.5% of women with AT deficiency, 28.0% with PC deficiency and 13.2% with PS deficiency developed thrombosis in the peripartum period of the current pregnancy, showing significant differences compared to the control data for pregnant women (*p* < 0.0001) (Table 4). Pregnant women with AT and PC deficiency were more susceptible to thrombosis than those with PS deficiency (*p* < 0.01). Of the thromboses that occurred, 92.3% occurred during pregnancy, and 62.8% occurred at less than 15 GW (Fig. 1). As the earliest onset of thrombosis was 5 GW, prophylactic anticoagulation should be started as early as pregnancy is confirmed in women with previously diagnosed thrombophilia. Nine cases of thrombosis (5 cases of AT deficiency, 3 cases of PC deficiency and one case of PS deficiency) occurred despite prophylactic anticoagulation and in all of these cases there was a previous history and/or family history of thrombosis. On the other hand, 69 cases of thrombosis occurred in women who did not receive prophylactic anticoagulation, 25 of whom had a previous history and/or family history of thrombosis. Prophylactic anticoagulation significantly prevented the onset of peripartum thrombosis (*p* < 0.0001) (Table 5). Among 6 cases in which thrombosis developed postpartum, the latest occurrence was 3 months after delivery. Therefore, postpartum prophylactic anticoagulation for at least 3 months should be considered. The most commonly used prophylactic anticoagulation was 10,000 units/day of UFH. In the antepartum period, LDA alone without UFH, although it is not an anticoagulant therapy, was used in 90 cases (3 cases of AT deficiency, 6 cases of PC deficiency and 81 cases of PS deficiency) (Table 5). This seems to be the reason that prophylactic treatment is considered for RPL.

RPL is a state in which a woman has two or more consecutive abortions [32, 33]. As a hypercoagulable state is associated with RPL and sterility of unknown causes, screening for thrombophilia should perhaps be considered. A meta-analysis of 89 observational studies reported that women with FV Leiden mutation, prothrombin G20210A mutation, or PS deficiency had higher risks of RPL, and that AT or PC deficiencies were not associated with increased risk of RPL when compared with the reference population [34]. However, there is a large and contradictory literature on the association between maternal hereditary thrombophilia and RPL [32, 35–45]. Although the measurement of factor XII (FXII) is meaningful [43, 46], there was no significant difference in pregnant women with AT, PC, or PS deficiency [2]. With regard to decreased PS activity, an acquired decrease due to PS antibodies rather than to a genetic predisposition has been suggested [47]. In this survey, the rate of RPL was significantly higher in women with PS deficiency than in the controls (*p* < 0.0001) (Table 2). Considering the many published reports, although an association between low PS activity and RPL was demonstrated in this survey, it is unknown whether it is related to hereditary PS deficiency. In women with PC deficiency, the rate of RPL was significantly higher than in the controls in this survey (*p* < 0.0001). Although anecdotal evidence of high fetal loss has been reported in cases of severe PC deficiency [48], the reason for this association is unknown. A large-scale prospective study is needed.

In the current pregnancies in this study, obstetrical information such as age, body mass index, GW at delivery and preterm delivery were not significantly different compared to controls, but the rates of C-section, especially emergent C-section, in women with PC deficiency only were significantly higher (*p* < 0.05) than in controls. The reason why the rate of C-section in women with PC deficiency was 50% is unclear, but greater attention to the mode of delivery may be necessary in cases of PC deficiency (Table 3). Two cases of spontaneous abortion in women with AT deficiency (13 WG, 19 WG), one case in a woman with PC deficiency (19 GW) and one case in a woman with PS deficiency (20 GW) were observed. These data might suggest no association with RFL.

Fetal/neonatal risk for fetal demise, growth restriction, preterm birth, perinatal stroke, and cerebral palsy have been reported in infants with inherited thrombophiliac variants [16, 17]. In this survey, though there were significant increases in premature rupture of the membranes, placenta previa/accreta in women with PS deficiency and intrauterine growth restriction (SGA infants) in women with AT deficiency, there were no significant differences in the abovementioned maternal or fetal/neonatal complications due to placental insufficiency related to a hypercoagulable state compared to controls. On the contrary, gestational diabetes mellitus, atonic bleeding, breech presentation, multiple pregnancy, non-reassuring fetal status and neonatal asphyxia were significantly lower than in controls (Tables 3, 4 and 7).

In AT deficient pregnant women, administration of 1500 to 3000 units of AT concentrate (1.2 times the dosage with recombinant preparations) is recommended as supplemental therapy in addition to basic UFH, so that AT activity is at least 70% [1, 49, 50]. In this survey, prophylactic AT supplementation (the abovementioned recommended units) in patients with AT deficiency was more frequently performed compared to prophylactic anticoagulation both in the antepartum (54 cases vs. 42 cases) and intrapartum (48 cases vs. 19 cases) periods. Conversely, prophylactic anticoagulation was used more than AT supplementation (72 cases vs. 29 cases) in the postpartum period (Tables 5 and 6). Judging from the single case of postpartum thrombosis in a woman with AT deficiency, the obtained data seem reasonable. Accordingly, both prophylactic AT supplementation and prophylactic anticoagulation might be suggested in the antepartum period, AT supplementation rather than anticoagulation might be suggested in the intrapartum period, and anticoagulation rather than AT supplementation might be suggested in the postpartum period.

This study has several limitations. In this survey, pregnant women with hereditary thrombophilia were selected according to our inclusion and exclusion criteria. Pregnant women who did not meet the inclusion criteria, or who did meet the exclusion criteria, were excluded from this study. In particular, women with low PS activity measured only during pregnancy had to be excluded from the PS deficiency group. However, genetic screening was not performed in most cases. Although an association between low PS activity and RPL was demonstrated in this survey, it is unknown whether this is related to hereditary PS deficiency. The control data we used in this survey do not necessarily reflect the average Japanese data for delivery, because these data were collected from general hospitals and perinatal medical centers that tended to manage high-risk deliveries throughout Japan.

## Conclusions

In conclusion, this survey revealed the thrombosis-related characteristics of pregnant women with hereditary thrombophilia in Japan. The number of pregnancies in women with AT, PC and PS deficiency might be as many as 27, 17 and 108, respectively, every year in Japan. Pregnant women with AT and PC deficiency were more susceptible to thrombosis than those with PS deficiency. Of the thromboses that occurred, 92.3% occurred during pregnancy and 62.8% occurred at less than 15 GW. Prophylactic anticoagulation significantly prevented the onset of peripartum thrombosis. It is unknown whether RPL with low PS activity is related to hereditary PS deficiency. In this survey, there seem to have been few serious maternal or fetal/neonatal complications due to placental insufficiency related to a hypercoagulable state other than growth restriction.

## Data Availability

The data that support the findings of this study are available from the authors but restrictions apply, as these data were used under license from the JSOG for the current study, and so are not publicly available. Data are, however, available from the authors upon reasonable request and with permission from the JSOG.

## Abbreviations

AT: Antithrombin
DVT: Deep vein thrombosis
FV: Factor V
GW: Gestational weeks
HDP: Hypertensive disorder of pregnancy
JSOG: Japan Society of Obstetrics and Gynecology
LDA: Low dose aspirin
LMWH: Low molecular weight heparin
PC: Protein C
PE: Pulmonary embolism
PS: Protein S
RPL: Recurrent pregnancy loss
SGA: Small for gestational age
UFH: Unfractionated heparin
VTE: Venous thromboembolism
